# Ophthalmic Self-Medication Practices and Associated Factors of Using Steroid Eye Drops Among Adult Ophthalmic Patients

**DOI:** 10.7759/cureus.43110

**Published:** 2023-08-08

**Authors:** Sarah S Alamer, Shujon M Alazzam, Amjad K Alanazi, Mohamed A Sankari, Jana S Sendy, Amani E Badawi, Khalid Allam, Saleh A Alkhaldi

**Affiliations:** 1 Department of Medicine, Almaarefa University, Riyadh, SAU; 2 Department of Medicine, Mansoura University, Mansoura, EGY; 3 Department of Ophthalmology, King Saud Medical City, Riyadh, SAU; 4 Department of Research Center, King Saud Medical City, Riyadh, SAU

**Keywords:** over the counter, steroid eye drop, ophthalmology, ophthalmic medicine, self-medication

## Abstract

Background: Self-medication is defined as the selection of medicines by individuals to treat self-diagnosed ophthalmic symptoms without consultation of healthcare professionals. Topical steroids can produce severe eye-threatening complications, including the elevation of intraocular pressure (IOP) with possible development of glaucoma and infrequent optic nerve damage. In recent times, many over-the-counter (OTC) ophthalmic preparations are possible without a prescription. There are a lot of concerns about the safety of long-term use of nonprescription ophthalmic drugs, which may lead to a variety of serious ocular complications.

Objective: To determine the prevalence of self-medication ocular topical steroid practices and associated factors among adult ophthalmic patients attending ophthalmology clinics at King Saud Medical City (KSMC) in Riyadh.

Methods: A cross-sectional study targeted adults aged 18 or older who had used topical steroids eye drops. The data were collected through interviews, and an online questionnaire was distributed among patients who attended ophthalmology clinics between December 2022 and February 2023.

Result: From a total of 308 responses, 92 (29.8%) were using ocular topical, 58 (18.8%) with a prescription, five (1.6%) without a prescription, and 29 (9.4%) with and without a prescription, while 216 (70.1%) did not use it. The frequency of using ocular topical steroids without a prescription among participants was 11 (12%) once and 33 (35%) many times. Additionally, 26 (28.3%) were having complications, mostly eye infections (11, 12.4%), glaucoma (8, 9%), and cataracts (6, 6.7%). The reasons for practicing self-medication of steroid eye drops among participants were 14 (15.2%) repeated symptoms, 11 (15.2%) had heard advice from friends, and 11 (15.2%) think they had enough knowledge.

Conclusion: The study reported the use of self-medication with steroids in ophthalmology clinics at KSMC, even though detecting a high level of perception and acceptable practices among participants. This practice is mainly due to participants having repeated symptoms and thinking they have enough knowledge. Educating the patients would help in reducing the incidence of self-medication steroid eye drops and its associated complications.

## Introduction

Self-medication is defined as the selection and use of medicines by individuals (or a member of an individual's family) to treat self-recognized or self-diagnosed ophthalmic conditions or symptoms without consultation of healthcare professionals [1-2]. According to WHO’s definition, “self-medication involves the use of medicinal products by the consumer to treat self-diagnosed disorders or symptoms, or the intermittent or continued use of medication prescribed by a physician for chronic or recurrent diseases or symptoms" without consultation of healthcare professionals [3-4]. Inappropriate self-medication results in the irrational use of drugs, wastage of resources, and increased resistance to pathogens, which entails serious health hazards, such as adverse reactions and prolonged suffering [5]. This behavior includes buying medicines without a prescription, using leftover doses from previous prescriptions, sharing medicines with other family members or social groups, or misusing the medical prescription either by prolonging, interrupting, or modifying the dosage and the administration period [6]. When patients do not instill eye drops correctly, their clinical outcomes can be negatively affected [7]. Although over-the-counter (OTC) drugs are meant for self-medication and are of proven efficacy and safety, their improper use due to a lack of knowledge of their side effects and interactions could have serious implications, especially in extremes of ages (children and old age) and special physiological conditions, such as pregnancy and lactation [8]. Self-medication practice is a common phenomenon all over the world, including in industrialized and developing countries [9-10]. The prevalence of irresponsible self-medication is high all over the world [11]. A continuous worldwide increase in self-medication has been triggered by economic, political, and cultural factors, and the practice is becoming a major public health problem [3]. Traditional eye medicine (TEM) is a form of biologically based therapies, practices, or partially processed organic or inorganic agents that can be applied through different routes of administration to achieve a desired ocular therapeutic effect, associated with various ocular complications, including blindness [11]. Corticosteroid has been used in ophthalmology for almost 70 years[ [12]. Corticosteroid-containing preparations are involved in acute anterior uveitis and perforation of the globe [13]. Keratitis, conjunctivitis, corneal ulcers, mydriasis, conjunctival hyperemia, loss of accommodation, and ptosis have occasionally been reported following local use of corticosteroids [12-13]. These steroid medications are easily available at low cost without prescription at various pharmacy shops and are often prescribed by pharmacists [9]. However, the misuse of self-medication costs money and wastes resources [14]. Topical steroids can produce severe eye-threatening complications, including, elevation of IOP with possible development of glaucoma and infrequent optic nerve damage, posterior subcapsular cataract formation, and delayed wound healing [12]. In recent times, many OTC ophthalmic preparations are possible without a prescription. Antihistamine, decongestant, and lubricating eye drops are among the available OTC ophthalmic medication list. A study from India done to determine the level of awareness in public to OTC ophthalmic preparations was shown that 99.1% of people lack knowledge about two types of ophthalmic preparations, OTC eye drops and prescription-only eye drops, and reported three cases of total vision loss, as a result of abuse of OTC eye drops with a history of angle closure glaucoma [15]. A study in Saudi Arabia reported that the rate of self-medication with prescription drugs (topical antibiotics and steroid-containing eye drops) was 37.2%. Among 1,354 subjects, 662 were males (49%), and 692 were females (51%) [16]. There are a lot of concerns about the safety of long-term use of nonprescription ophthalmic drugs, which may lead to a variety of serious ocular complications [15]. Eye diseases are common in patients residing in long‐term care facilities, and ophthalmic solutions are frequently prescribed [17]. A study in Bengal reported that it has been observed that a majority of rural patients attending the eye outpatient department (OPD) in a tertiary care hospital at Rampurhat, West Bengal, are in the habit of misusing steroid medications through self-medication, pharmacists, and general practitioners for various eye conditions, which can lead to various complications in eyes [13]. The aim of the current study is to determine the prevalence of self-medication ocular topical steroid practices and associated factors among adult ophthalmic patients attending ophthalmology clinics at King Saud Medical City (KSMC), Riyadh City.

## Materials and methods

A cross-sectional study, targeting participants aged 18 years old or above who had used topical steroids eye drops to determine the prevalence of self-medication ocular topical steroid practice and associated factors among adult patients attending ophthalmology clinics at KSMC. The study was approved by the Research Ethics Committee of KSMC in Riyadh and was conducted in accordance with the tenets of the Declaration of Helsinki for research involving human participants and in line with current legislation on clinical research. All participants completed a consent form for participation prior to the start of the study after a verbal explanation about the study. A total of 308 participants were recruited. The data were collected through interviews and an online questionnaire distributed among patients attending ophthalmology clinics between December 2022 and February 2023. The questionnaire consists of a paragraph explaining the purpose of the study, the benefits of participation, the right to withdraw at any point, and requesting their voluntary participation by answering the questionnaire.

Statistical analysis

The data were analyzed through the Statistical Package for the Social Sciences version 25 (SPSS Inc., Chicago, IL, USA) for Windows. The results were presented in tables as frequencies and percentages. Suitable statistical tests of significance were used to determine the results, and a p-value of less than 0.05 is considered significant. 

Ethical considerations

The ethical approval of the IRB in Almaarefa University College of Medicine was fulfilled before the start of data collection, and the aim of the study was explained clearly to the participants. The ethical approval of the research ethics committee of KSMC in Riyadh was fulfilled before the start of data collection and conducted in accordance with the tenets of the Declaration of Helsinki for research involving human subjects and current legislation on clinical research. All participants completed a consent form for participation prior to starting the study. Patients were asked about their availability and willingness to participate in the survey, and they understand, through a written informed consent form, that participation in the study is voluntary and will not influence their wait time before consultation; they were also informed that their refusal to participate will not negatively affect their medical care. The data will be kept confidential. Only the researchers will have access to the database only for analysis purposes.

## Results

A total of 308 responses were received, 174 (56.5%) were females, and 134 (43.5%) were males. The majority of the respondents (228, 74%) were younger than 38 years old, and a few of them were older than 39 years old (25.9%). See Table 1 for more details.

**Table 1:** Demographic data of the subjects.

Variable		Frequency	Percent
Age	18-28	111	36%
	29-38	117	38%
	39-48	46	14.9%
	≥49	34	11%
Social Status	Married	179	58.1%
	Single	129	41.9%
Nationality	Saudi	300	97.4%
	Non-Saudi	8	2.6%
Gender	Female	174	56.5%
	Male	134	43.5%
Education Level	Less than high school	20	6.5%
	High school	54	17.5%
	University	96	31.2%
	Postgraduate	138	44.8%
Occupation	Government sector	143	46.4%
	Private sector	8	2.6%
	Unemployed	60	19.5%
	Student	97	31.5%
Total		308	100%

Most of the participants had a good level of education; 74.4% visited ophthalmologists to get a prescription, 25.6% thought that it is not important to visit ophthalmologists to get a prescription, 64.6% thought that ocular topical steroid should be under prescription, and 35.4% thought it is should not be under prescription. Meanwhile, 64.4% thought that using topical cortisone eye drops without consulting a doctor may cause complications, and 35.6% thought that it does not cause complications (Table 2).

**Table 2:** Knowledge of participants about the importance of visiting ophthalmologists to get a prescription, following up during using the steroid, and its complications.

Question	Level of knowledge			
	Good		Poor	
	Frequency	Percentage	Frequency	Percentage
Do you think visiting an ophthalmologist to get a prescription is important?	229	74.4%	79	25.6%
Do you think that an ocular topical steroid should be under prescription?	199	64.6%	109	35.4%
Do you think that using topical cortisone eye drops without consulting a doctor may cause complications?	197	64.4%	109	35.6%

Among the 308 responses, 92 (29.8%) were using an ocular topical steroid, 58 (18.8%) using an ocular topical steroid with a prescription, five (1.6%) using an ocular topical steroid without a prescription, and 29 (9.4%) using an ocular topical steroid with and without prescription, while 216 (70.1%) did not use it. Only 14 responses (15.4%) were not following up with ophthalmologists while using an ocular topical steroid, 28 (30.8%) claimed sometimes, and 49 (53.8%) were following up. The frequency of using an ocular topical steroid without a prescription among responses were 11 (12%) once, 33 (35%) many times, and 48 (52%) did not use it without a prescription (Table 3).

**Table 3:** Patients follow up with ophthalmologists while using an ocular topical steroid and the frequency of using an ocular topical steroid without a prescription.

Question		Frequency	Percentage
Have you ever used an ocular topical steroid?	Yes, with prescription	58	18.8%
	Yes, without prescription	5	1.6%
	Yes, with and without prescription	29	9.4%
	I did not use it	216	70.1%
Do you usually follow up with your ophthalmologist while you are using an ocular topical steroid?	Yes	49	53.8%
	No	14	15.4%
	Sometimes	28	30.8%
How many times have you used an ocular topical steroid without a prescription?	Once	11	12%
	Many times	33	35%
	I did not use it without prescription	48	52%

The incidence of complications for self-medication practice of an ocular topical steroid was 71.7% for those who did not use an ocular topical steroid, 28.3% for those who had complications such as 11 (12.4%) due to eye infection, eight (9%) due to glaucoma, six (6.7%) due to cataracts, six (6.7%) for others, and majority 58 (65.2%) were having no complications (Table 4).

**Table 4:** Incidence of complications for self-medication practice of an ocular topical steroid among participants.

		Frequency	Percentage			Frequency	Percentage
Have you ever had any complications when you use an ocular topical steroid?	Yes	26	28.3%	If yes, what are the complications that you have?	Eye infection	11	12.4%
					Glaucoma	8	9%
					Cataracts	6	6.7%
					Others	6	6.7%
					No complication	58	65.2%
	No	66	71.7%				

**Figure 1:**
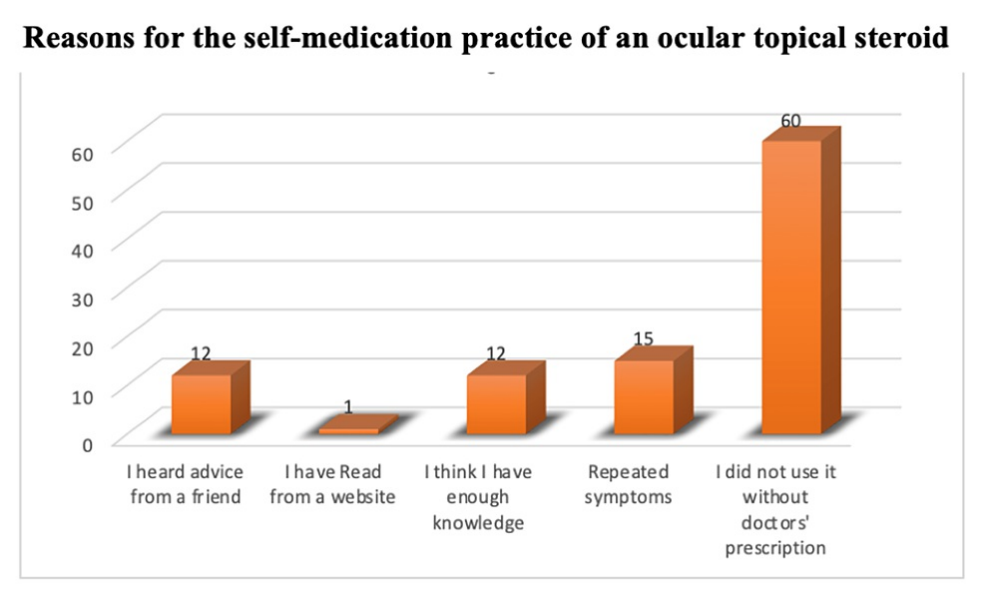
Reasons for the self-medication practice of an ocular topical steroid.

Reasons for self-medication practice of ocular topical steroid among participants were 14 (15.2%) repeated symptoms, 11 (15.2%) had heard a piece of advice from a friend, 11 (15.2%) think they had enough knowledge, and one (1.1%) has read from a website. Additionally, 55 (59.8%) did not use it without a doctor's prescription (Figure 1).

**Figure 2:**
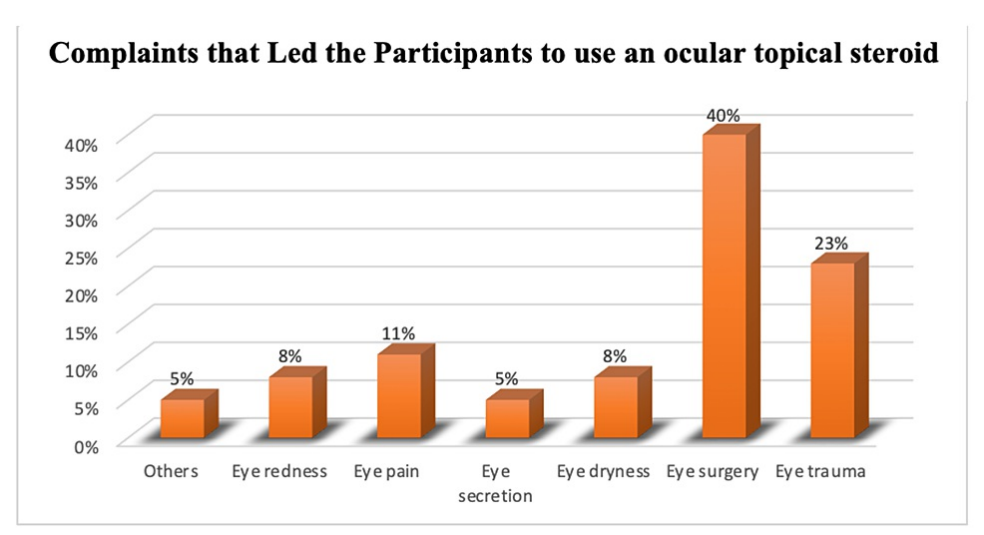
Complaints that led the participants to use an ocular topical steroid.

Complaints that led the participants to use an ocular topical steroid were 36 (39.6%) due to eye surgery, 21 (23.1%) due to eye trauma, 10 (11%) due to eye pain, seven (7.7%) due to eye redness and dryness, five (5.5%) due to eye secretion, and others (Figure 2).

## Discussion

In the present study, most of the participants had a good level of education. Specifically, 74.4% visited ophthalmologists to get prescriptions, 25.6% thought that it was not important to visit ophthalmologists to get prescriptions, 64.6% showed that an ocular topical steroid should be prescribed, and 35.4% thought that it should not. This is not consistent with an Indian study, where 56 out of 75 patients reported using eye drops without proper consultation [7]. Our study shows that 64.4% of participants think that using topical steroid eye drops without a proper consultation may cause complications, and 35.6% think that using topical steroid eye drops without consulting a doctor may not cause complications. One study conducted in Saudi Arabia indicated that the majority of steroid users (86.6%) were not aware of any related ocular complications [13]. This difference is mainly related to the patient's knowledge and the care about the medication they use. Another research conducted in India reported a similar finding where 29% of the total study population used ophthalmic medications without proper consultation. [1] In general, the practice of self-medication for ophthalmic symptoms is apparent. Explaining to the public the possible health issues of practicing self-medication is critical. In this study, the incidence of complications for self-medication practice of ocular topical steroid was 71.7% for those who did not use ocular topical steroid, and 28.3% have had complication, such as eye infection (11, 12.4%), glaucoma (8, 9%), cataracts (6, 6.7%), and others (6, 6.7%). The majority (58, 65.2%), had no complications. A study was done in the Netherlands, of 88 patients (41 men and 47 women), one had transient ocular hypertension, one had optic disc cupping without any glaucomatous defects in his visual field, and seven were given the diagnosis of cataracts [6]. More attention should be offered to the education of people about the serious health complication that can occur from this uncareful use. Reasons for the self-medication practice of ocular topical steroids found in this study were repeated symptoms, advice from friends, personal feeling of having enough knowledge, and information from online websites, in agreement with a previous study [7]. The similarity between those studies indicates that patients are highly affected by their community in this matter, as well as by their knowledge of self-medication practice of ocular topical steroids. It is evident that most complaints led the participants to self-medication with ocular topical steroids, where 36 (39.6%) had eye surgery, 21 (23.1%) had eye trauma, 10 (11%) had eye pain, seven (7.7%) had eye redness and dryness, and five (5.5%) had eye secretion and others. In contrast, another study reported redness and itching (63%), pain (14%), watering from the eyes (15%), and trauma (8%) [9]. These differences could be related to the selection of study designs and target populations. The current study has some limitations, it included patients from a single healthcare center in the city. In addition, the study was restricted to participants using steroid eye drops. Future studies may benefit from including other facilities and investigating the use of other ocular treatments.

## Conclusions

Our study reveals the incidence of practicing self-medication with steroid eye drops despite detecting a high level of knowledge and perception among participants. This practice among participants was mainly due to having repeated symptoms and thinking they had enough knowledge. Increasing awareness of patients about self-medication of steroid eye drop practice and its associated complications will help reduce their incidence.
